# Total Hip Arthroplasty With Prophylactic Fixation of Greater Trochanter and Distal Femur in a Patient With a History of Multiple Myeloma and Breast Cancer

**DOI:** 10.7759/cureus.37971

**Published:** 2023-04-22

**Authors:** Tyler Small, Kevin Fox, Lauren Edge, John Harker

**Affiliations:** 1 Orthopaedic Surgery, Liberty University College of Osteopathic Medicine, Lynchburg, USA; 2 Orthopaedic Surgery, HCA Healthcare/USF Morsani College of Medicine GME: HCA Florida Largo Hospital, Largo, USA

**Keywords:** femoral lesion, distal femur, prophylactic fixation, multiple myeloma, total hip arthroplasty

## Abstract

Multiple myeloma is the most common primary malignancy of the bone marrow and may present as bone pain and/or pathologic fracture(s) in affected patients. Treatment of bone lesions typically consists of chemotherapy and radiation and may include prophylactic fixation in patients meeting specific criteria. This report reviews a case of a 74-year-old female with a history of multiple myeloma and breast cancer, previously treated with chemotherapy and radiation, who sustained a pathologic femoral neck fracture with associated ipsilateral lesions of the femoral shaft and peritrochanteric region. This patient received a total hip arthroplasty with a greater trochanteric claw plate and extended femoral stem for prophylactic fixation of the distal femur. In this report, the current literature surrounding the use of extended femoral stems for prophylactic fixation of femoral diaphyseal lesions will be reviewed and the above case will be presented. This case serves as a bridge between orthopedic oncology and arthroplasty as an extended femoral stem was used to prevent future pathologic fracture of distal femur lesions.

## Introduction

Multiple myeloma (MM) is the most common primary malignancy of the bone marrow and is most prevalent in males over the age of 40 [1]. Patients may initially present with bone pain and/or pathologic fracture(s), as well as fatigue secondary to anemia, renal insufficiency, and hypercalcemia, due to increased osteoclastic activity. Up to 14% of MM patients on bisphosphonate therapy sustain a pathologic fracture during their lifetime, which increases their risk of mortality by 28% [2,3]. MM involves a pathologic overproduction of plasma cells that leads to an overabundance of monoclonal immunoglobulins, as well as a variety of factors that both activate osteoclasts (RANK/RANKL) and suppress osteoblasts [4]. There is also a decrease in the production of Osteoprotegerin, which is crucial in the activation of osteoblasts. This imbalance of bone resorption and bone production is what leads to the typical punched out, or lytic lesions, associated with MM, which commonly involves the proximal femur (femoral head, femoral neck, and peritrochanteric region). The initial detection of these lesions is often made with plain radiographs of the affected area(s). Further evaluation of any lesions present can be conducted using cross-sectional computed tomography (CT) or whole-body magnetic resonance imaging (MRI), allowing for the identification of any bone marrow and/or soft tissue involvement. Definitive diagnosis of MM requires a bone marrow biopsy, while staging and treatment monitoring can be accomplished with the use of positron emission tomography (PET) scans. The mainstays of treatment for MM are chemotherapy and radiation to the affected areas, though prophylactic fixation of the diseased bone can be performed to address/prevent impending pathologic fractures.

There are multiple methods of predicting a patient’s risk of pathologic fracture that can guide decision-making toward prophylactic fixation. The Mirels’ Criteria is a numerical scoring system that is most used in predicting a patient’s risk of pathologic fracture. This scoring system is based upon four factors: the presence or absence of pain, location, type, and size of the bone lesion(s). A score of 8 or greater indicates an increased risk for pathologic fracture and warrants prophylactic fixation of the diseased bone. In patients where fixation is warranted, the location and type of lesion will determine the optimal fixation method. Historically, lesions of the femoral neck and head are treated with hemi or total hip arthroplasty (THA) while lesions of the peritrochanteric region and femoral shaft are stabilized with intramedullary nailing [5,6]. There is literature supporting prophylactic fixation of femoral shaft lesions in patients with incomplete or impending femoral shaft fractures using intramedullary nails [5]. Patients that undergo prophylactic fixation have been shown to bear weight sooner, and have decreased pain, and decreased mortality than those who do not [6-9]. There is a scarcity of information regarding the use of a total hip prosthesis with an extended modular femoral stem to prophylactically fixate the distal femur in a patient with an ipsilateral femoral neck fracture.

There is debate over whether stems implanted in oncologic patients undergoing hip arthroplasty should be cemented or press fit. While cementation of arthroplasty stems allows for more reliable fixation in patients with poor bone stock who may also be undergoing radiation, it is not without risk. Cemented stems carry a risk of cement embolization and bone cement implantation syndrome (BCIS), characterized by hypotension, hypoxia, cardiac arrhythmias, and cardiac arrest. Price et al. conducted a retrospective chart review of 42 patients who underwent THA with cemented long-stem femoral components to assess perioperative complications [10]. The perioperative complications found in this study included cement-associate hypotension (eight patients), prolonged intubation (five patients), and death (one patient), as well as oxygen desaturation, deep vein thrombosis, pneumonia, fracture, and progression of the disease. Baptista et al. also conducted a retrospective case series of 34 patients with metastatic bone disease who had undergone uncemented hip arthroplasties and ultimately concluded that those who received uncemented stems had lower rates of complications, earlier weight-bearing and a decreased length of hospital stay [11]. Larsen et al. came to a more neutral conclusion while retrospectively comparing outcomes in oncologic patients with press-fit implants to those with cemented implants [12]. It was found that there was no significant difference in 30-day mortality, postoperative complications, Harris Hip Scores, or Musculoskeletal Tumor Society Scores between patients who received a cemented stem vs an uncemented stem [12].

The use of a claw plate to reinforce the greater trochanter is well documented in revision THA and peritrochanteric fractures. A retrospective study of 41 patients was conducted by Tang et al. that evaluated the use of greater trochanteric claw plates in patients undergoing primary or revision THA with compromised greater trochanters [13]. It was found that patients who received the claw plate subjectively had a greater range of hip motion and overall function. These subjective improvements were attributed to improved stability and reduced shearing forces between the abductor muscles and the greater trochanter, allowing for more anatomic healing [13]. There is a paucity of data evaluating the use of a greater trochanteric claw plate for prophylactic fixation of the greater trochanter(s) in patients with oncologic lesions to the peritrochanteric region.

## Case presentation

This is a report of a 74-year-old, active Caucasian female with a history of breast cancer, MM, and osteopenia. She was diagnosed with HR+, HER2- breast cancer in 2015 and was immediately treated with a lumpectomy followed by Arimidex (anastrozole) chemotherapy. All margins and lymph nodes were negative at the time of surgery and she has remained on Arimidex since. Her MM was diagnosed near the end of 2017 and she began chemotherapy, with Revlimid (lenalidomide), in early 2018. She also underwent multiple rounds of radiation therapy between 2018 and 2021; her current chemotherapy agent is Darzalex (daratumumab). She takes Fosamax (alendronate) regularly for treatment of both her MM and osteopenia.

In Autumn 2021, months after her last round of radiation therapy, this unassisted community ambulator underwent a right THA for a pathologic basicervical femoral neck fracture that was initially detected on a PET scan. A right THA was performed using an extended femoral stem and in the presence of trochanteric lesions, a lateral claw plate was also used. She had an uneventful postoperative course that included formal physical therapy and was discharged on postoperative day four, ultimately returning to her baseline functional status.

**Figure 1:**
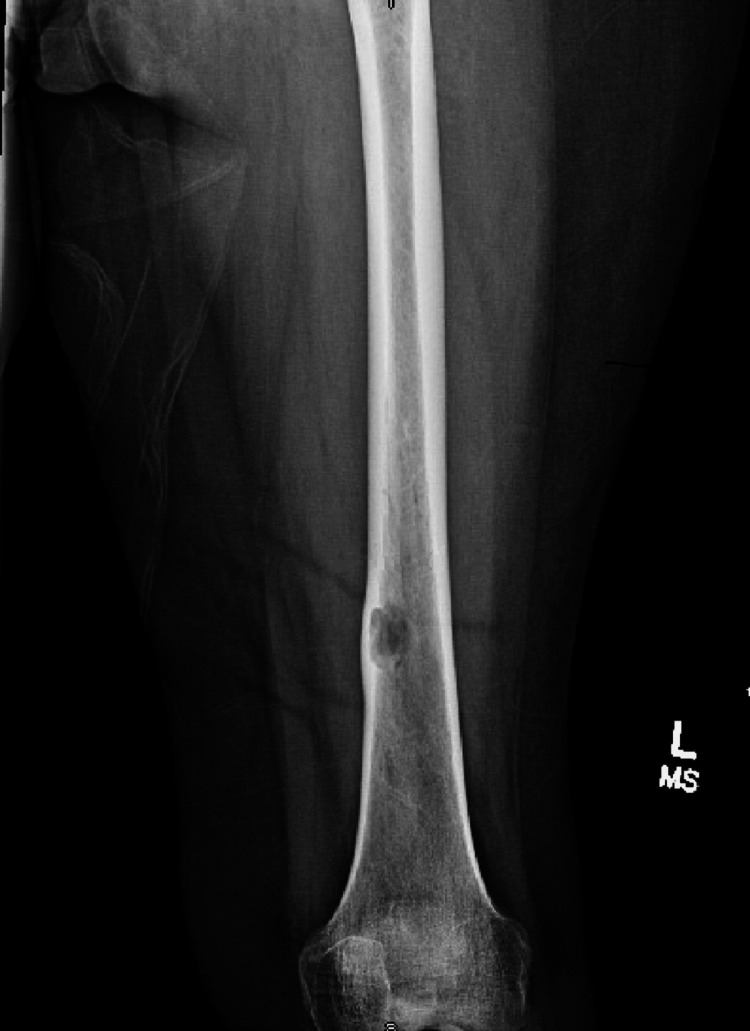
AP left femur demonstrating distal femur lesion AP: anteroposterior

**Figure 2:**
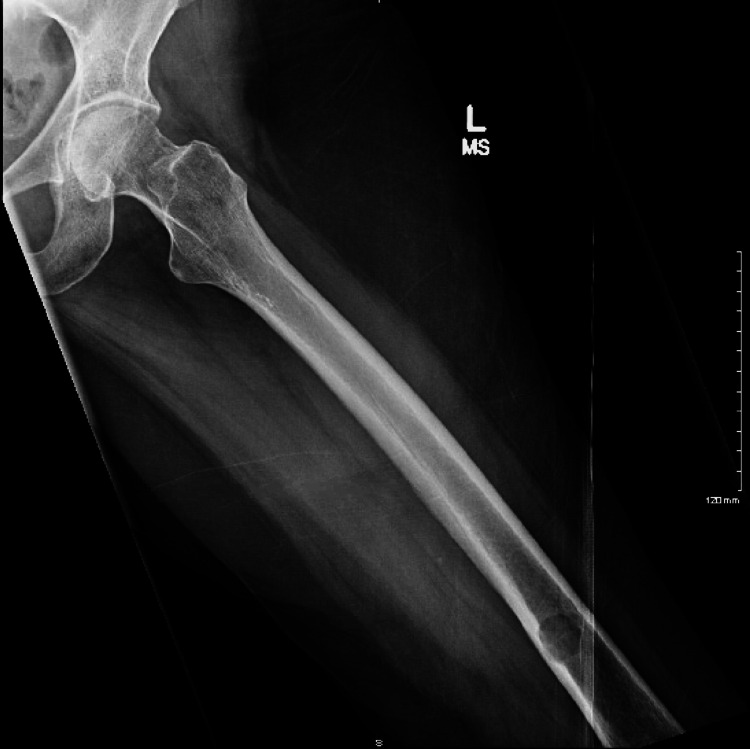
Lateral left hip demonstrating distal femur lesion

**Figure 3:**
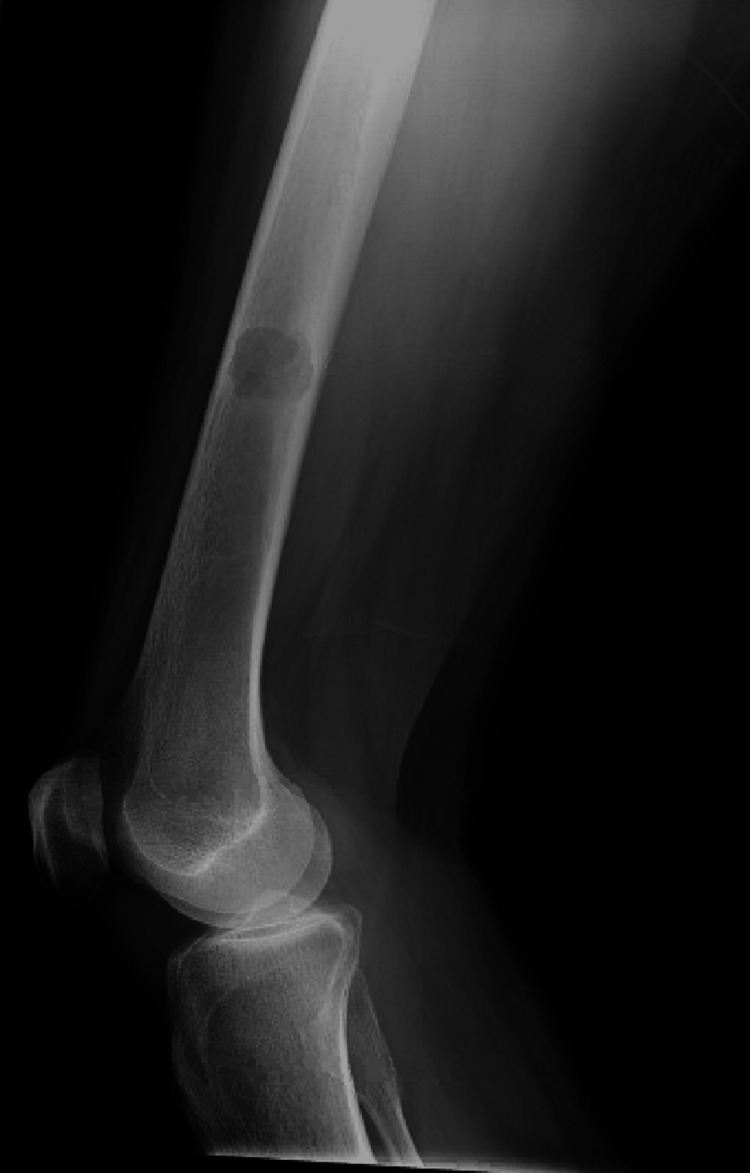
Lateral left knee demonstrating distal femur lesion

**Figure 4:**
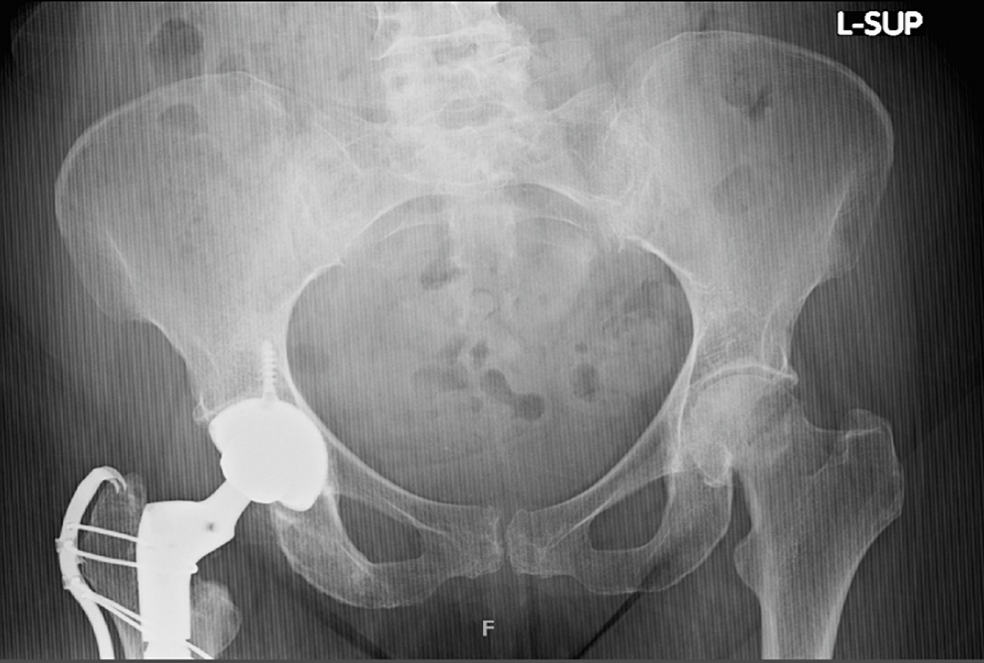
AP pelvis demonstrating left subcapital femoral neck fracture AP: anteroposterior

**Figure 5:**
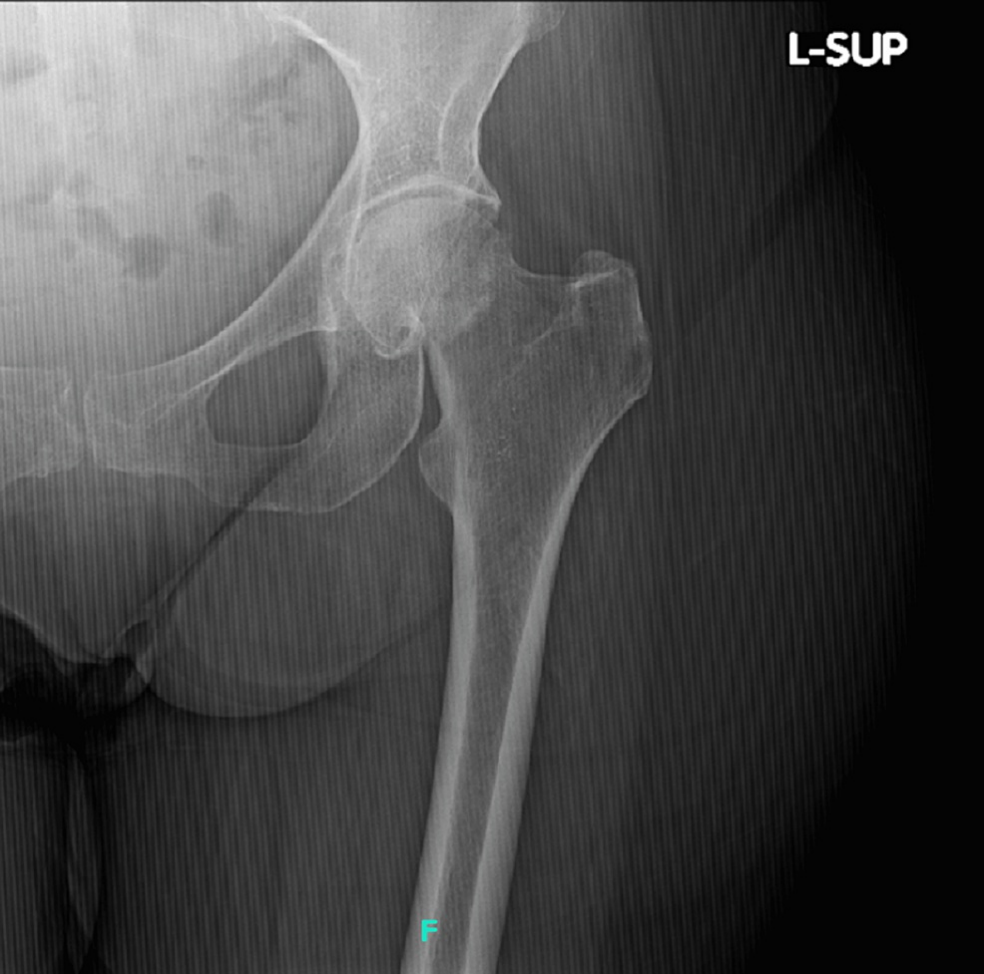
AP left hip demonstrating subcapital femoral neck fracture AP: anteroposterior

**Figure 6:**
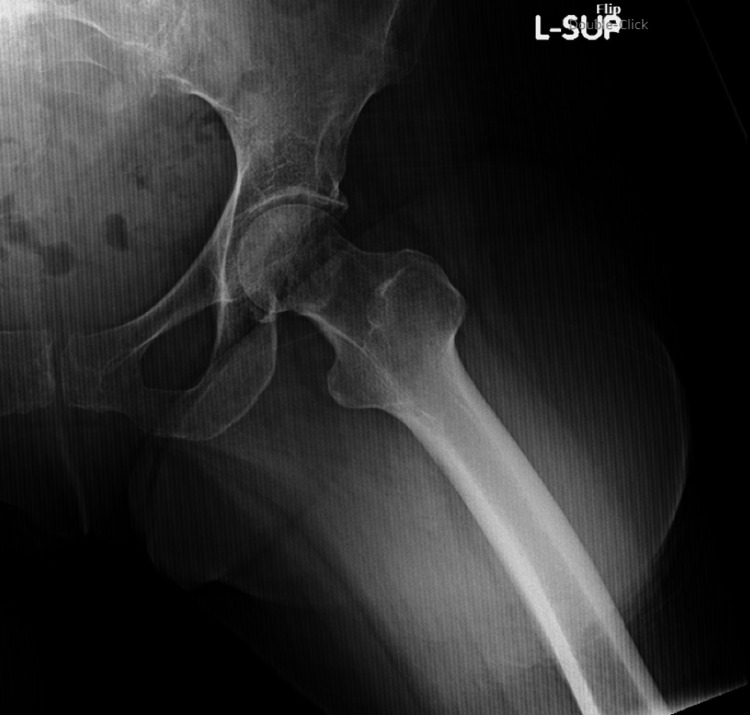
Lateral left hip demonstrating subcapital femoral neck fracture

**Figure 7:**
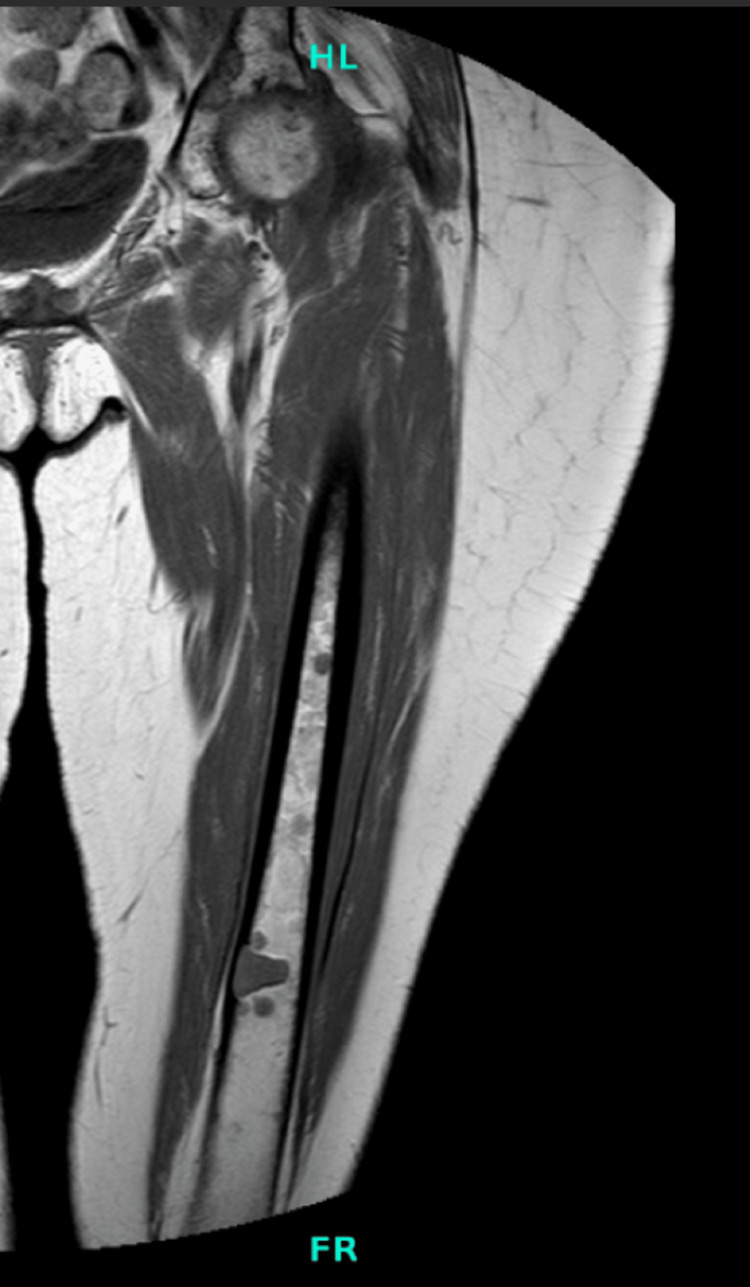
MRI of left hip demonstrating distal femur lesion MRI: magnetic resonance imaging

**Figure 8:**
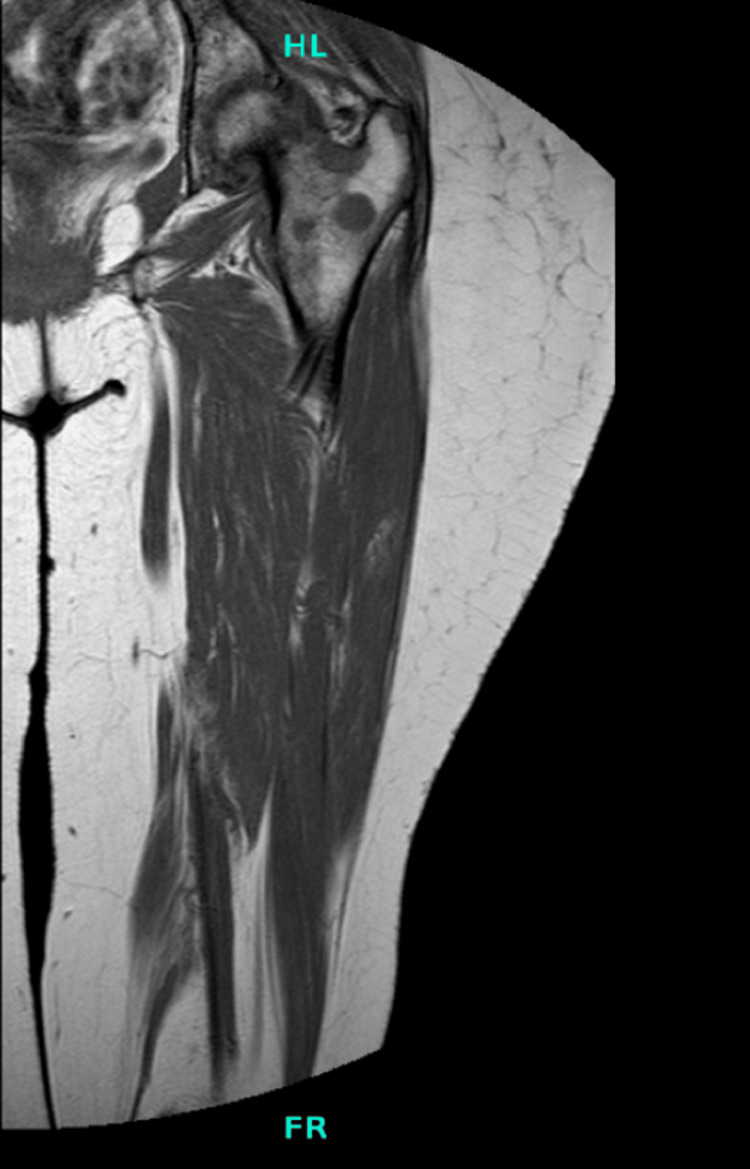
MRI of left hip demonstrating peritrochanteric lesions MRI: magnetic resonance imaging

Nine months after her index procedure on the right side, in early summer of 2022, this patient presented to the emergency department reporting an acute onset of left hip and thigh pain. Clinical evaluation and radiographs of the left hip were negative for acute fracture at that time; however, a small lytic lesion was noted in the left distal femoral diaphysis (Figures 1-3). A few weeks later, the patient presented to the clinic reporting increased left hip pain following a hiking trip, leaving her wheelchair-bound. She endorsed pain over the lateral aspect of her hip, groin, and anterior thigh. Radiographs demonstrated a pathologic subcapital femoral neck fracture (Figures 4-6). Additionally, an MRI of the left hip demonstrated the presence of peritrochanteric and distal femoral lesions (Figures 7-8).

**Figure 9:**
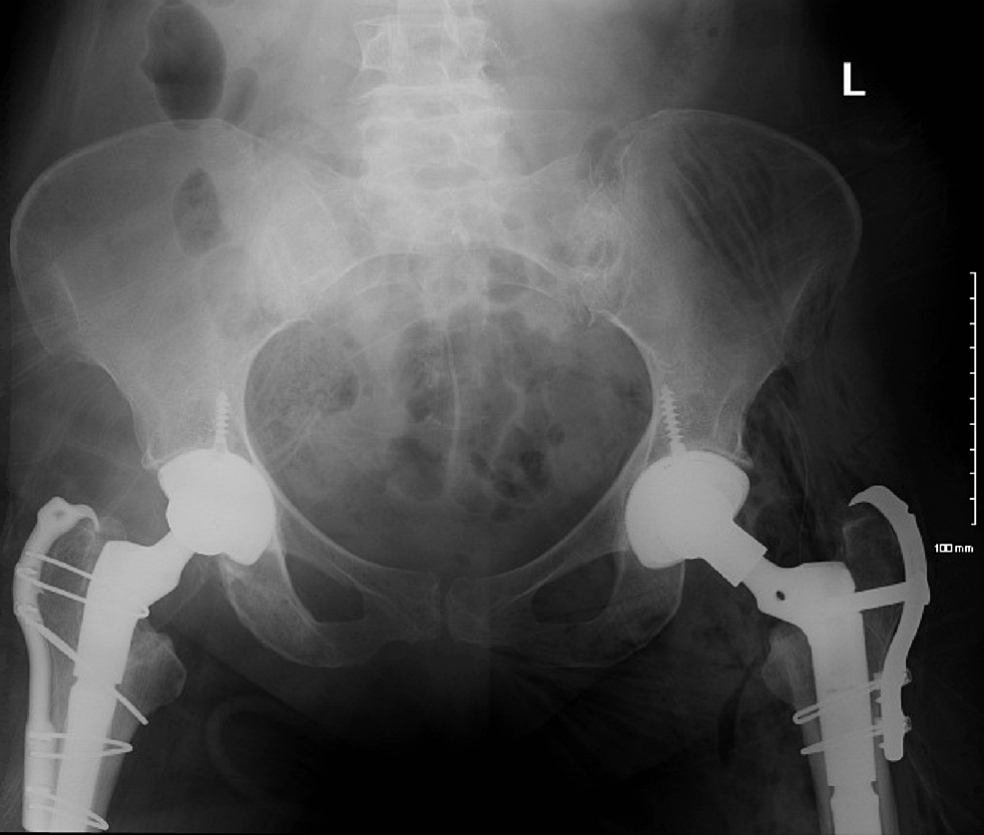
Postop AP pelvis AP: anteroposterior

**Figure 10:**
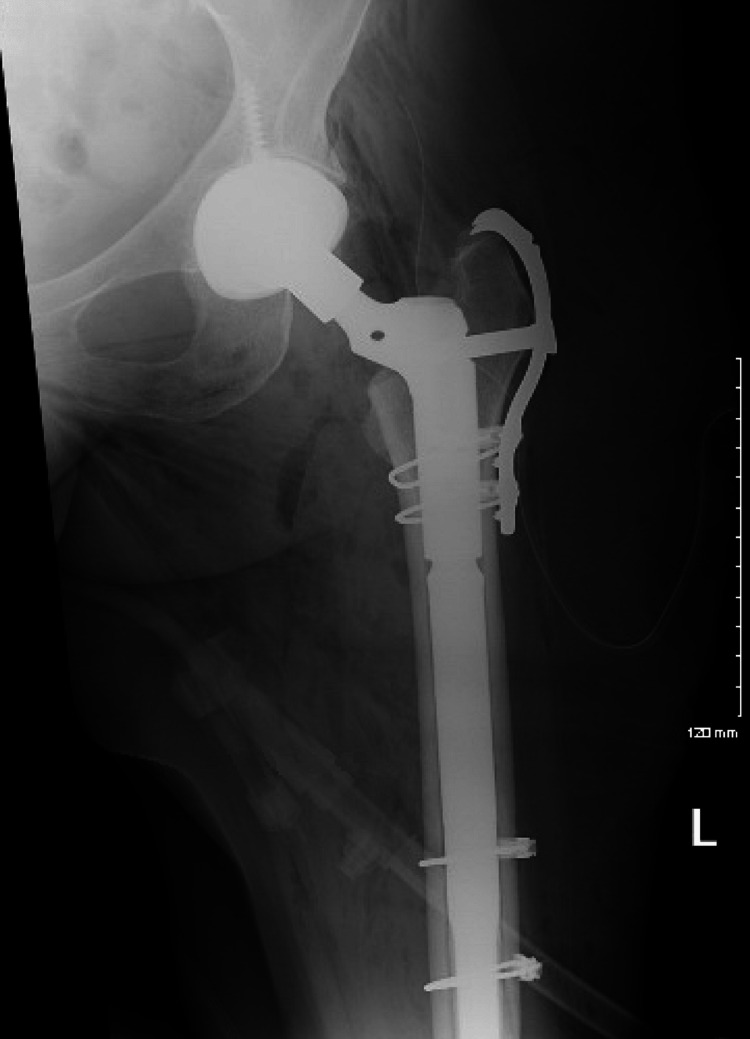
Postop AP left hip AP: anteroposterior

**Figure 11:**
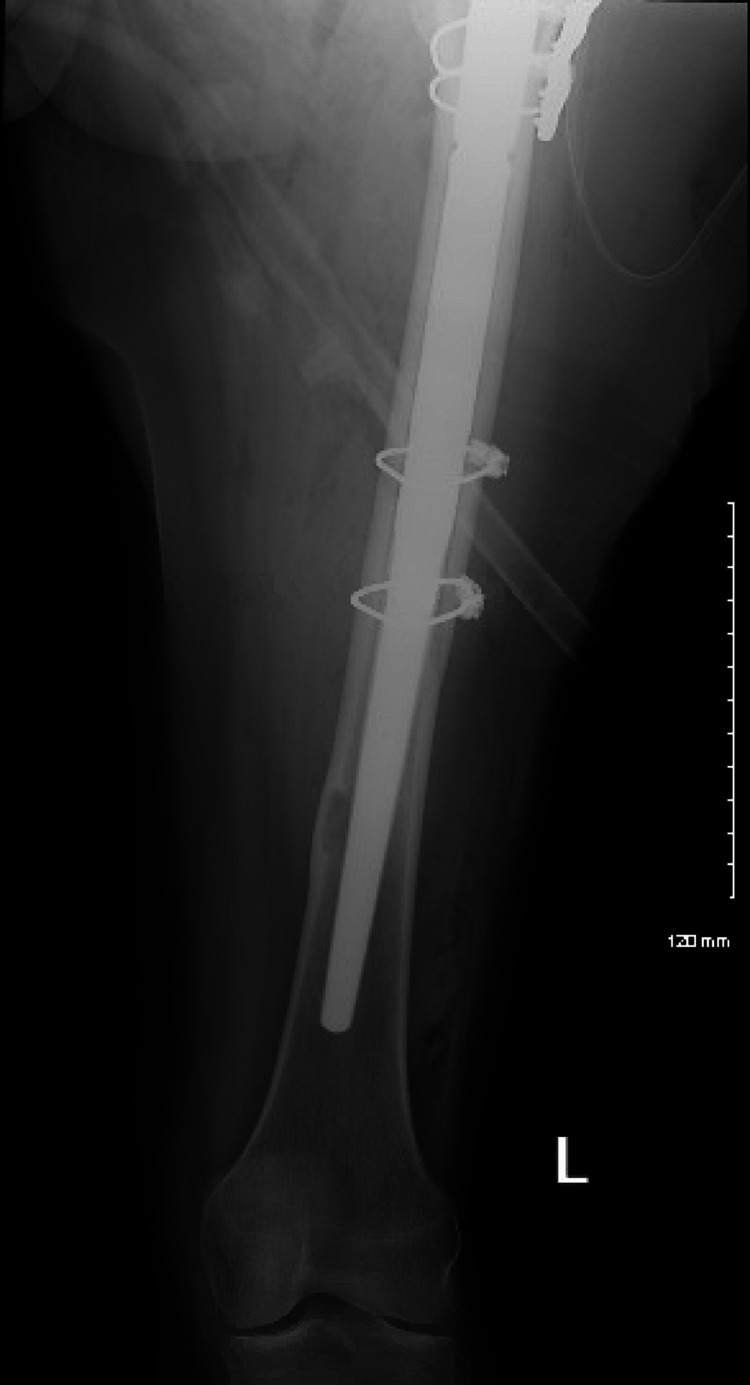
Postop AP left femur AP: anteroposterior

**Figure 12:**
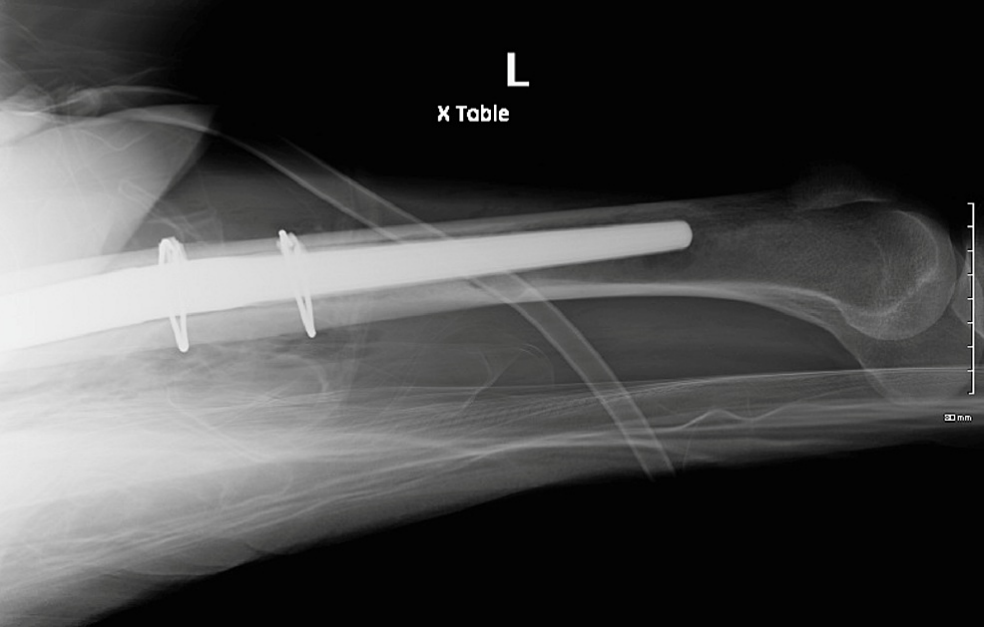
Postop lateral left femur

Given this patient’s high functional status, a left THA was chosen to treat her current fracture, over hemiarthroplasty. The bony lesions in the trochanteric region and distal femoral shaft all met the criteria for prophylactic fixation with scores of 8, so open reduction and internal fixation (ORIF) of the left greater trochanter and femoral shaft were planned for. She remained on her chemotherapy (Arimidex and Darzalex) and Fosamax regimen up until the time of surgery. Preoperative planning included the use of a lateral claw plate and extended femoral stem prosthesis to reinforce the trochanteric region and femoral shaft, respectively. A standard posterior approach to the hip was utilized for this procedure with the patient in the lateral decubitus position. Following acetabular preparation, a standard shell with single screw fixation and dual mobility acetabular liner were implanted. Given the use of revision femoral components, a more constrained acetabular component was used, adding more stability to the overall construct. The femur was then reamed and all reamings sent to pathology. The pathology report returned consistent with the patient’s known active MM without evidence of metastatic breast disease. A porous coated core proximal body with a modular head was utilized for the proximal modular femoral component. A bowed, uncemented, porous-coated distal stem was reamed for and implanted which was able to bypass the distal femur lesion by two cortical diameters. Femoral modular components were linked and impacted into place. Two cerclage cables were employed using a separate lateral thigh incision to reduce a midshaft femur fracture along the isthmus of the femur, which was sustained intraoperatively. Lastly, a trochanteric claw plate was used to prophylactically fixate the greater trochanter in the presence of peritrochanteric lesions. A trochanteric bolt and two cerclage cables were used to fixate the claw plate to the modular stem, creating a linked construct (Figures 9-12). The hip was reduced and noted to be stable through a range of motion and position of sleep.

**Figure 13:**
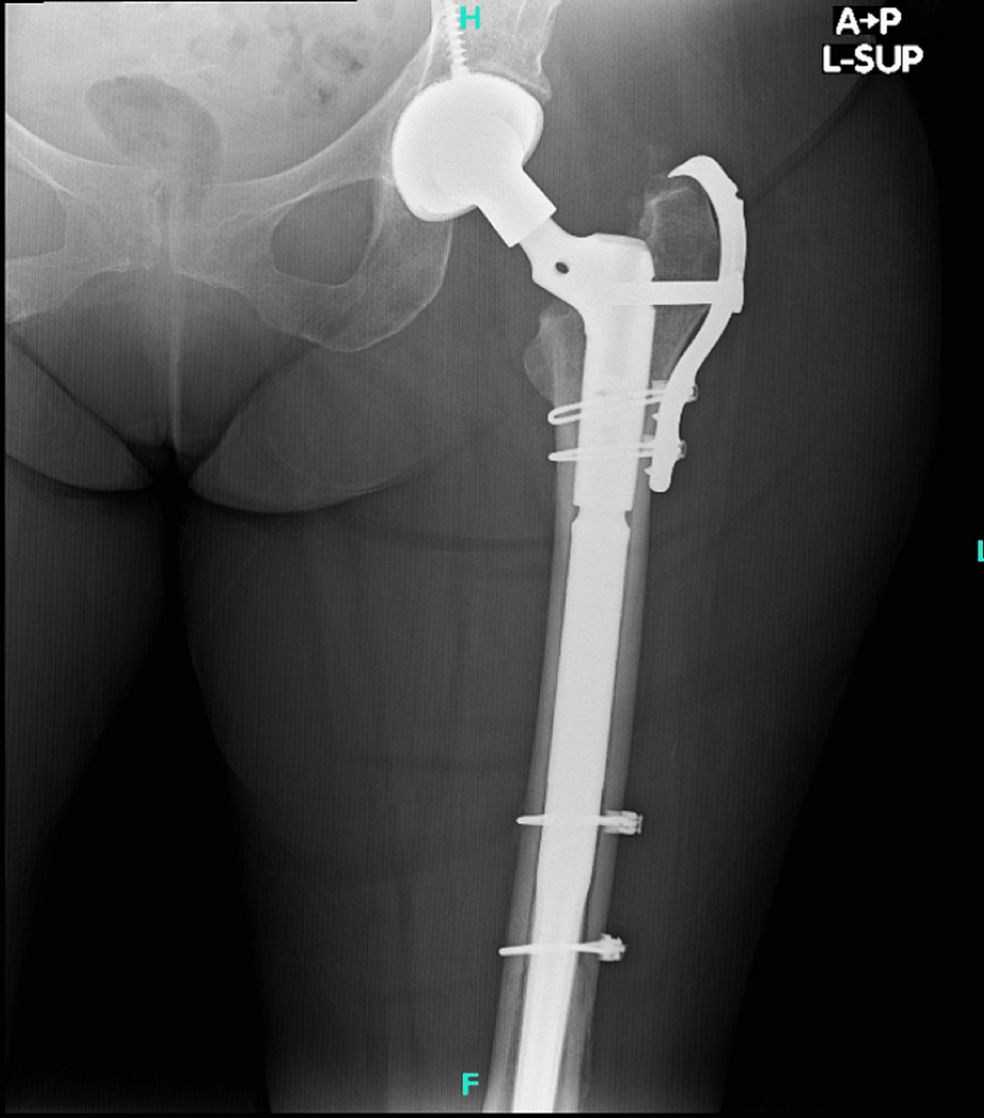
AP left hip at three-month follow-up AP: anteroposterior

**Figure 14:**
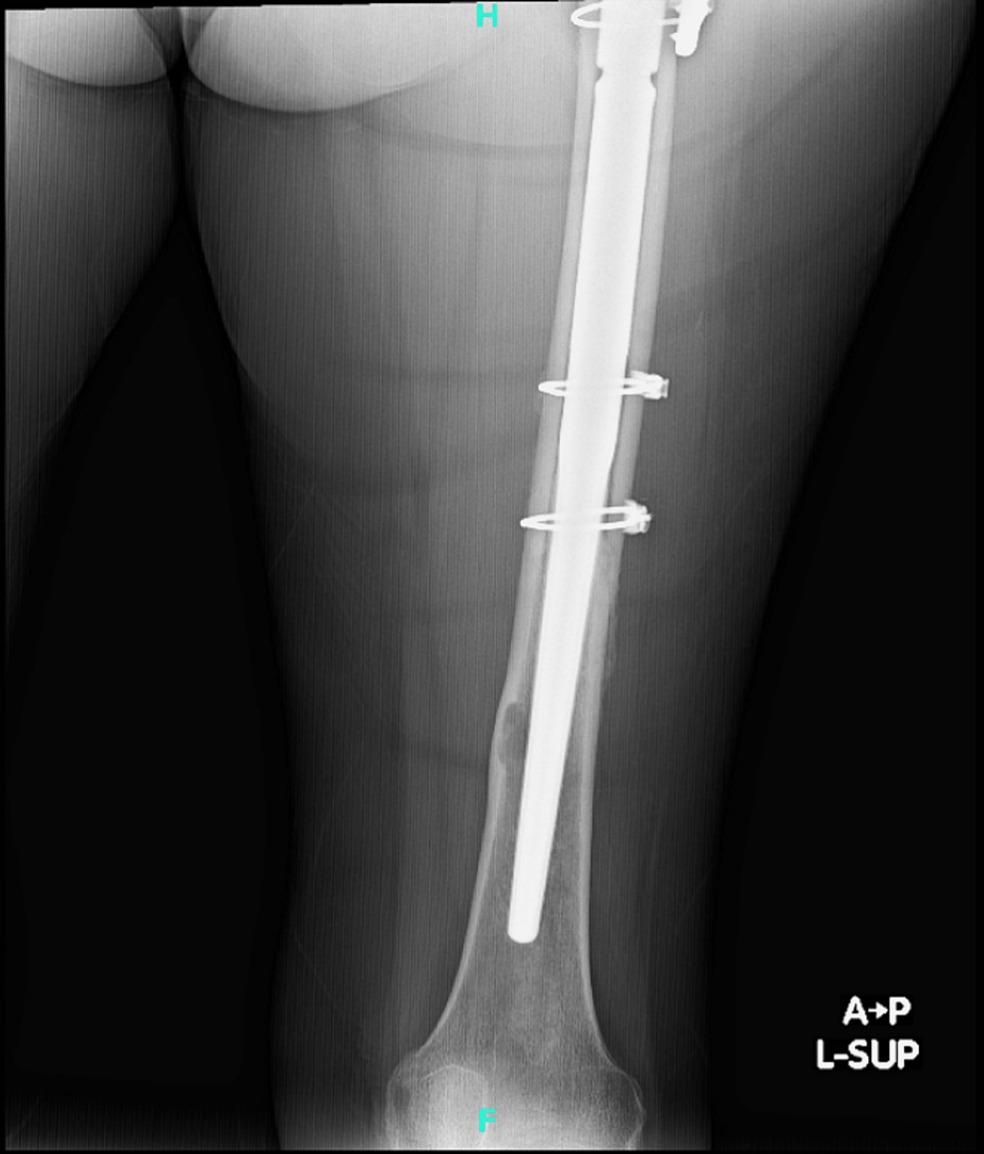
AP left femur at three-month follow-up AP: anteroposterior

**Figure 15:**
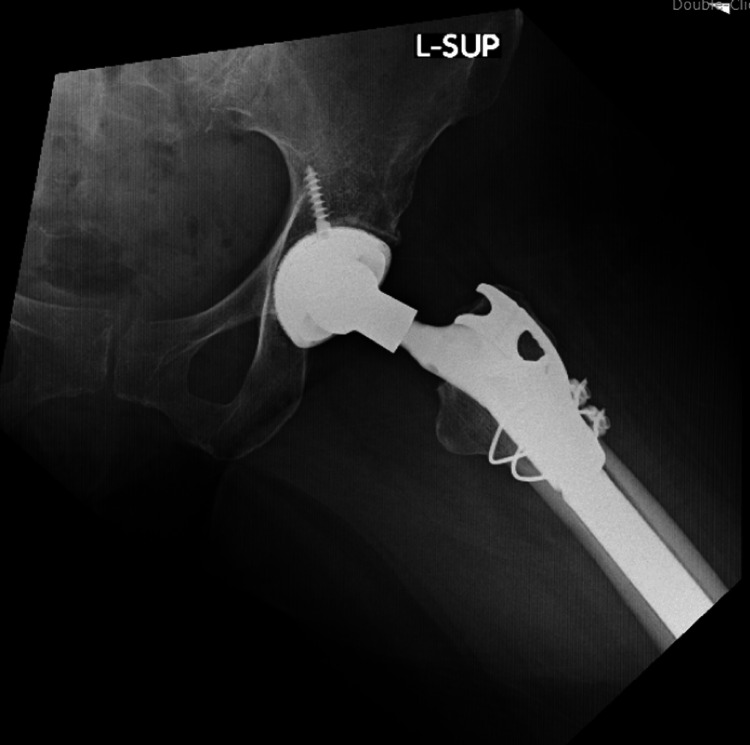
Lateral left hip at three-month follow-up

**Figure 16:**
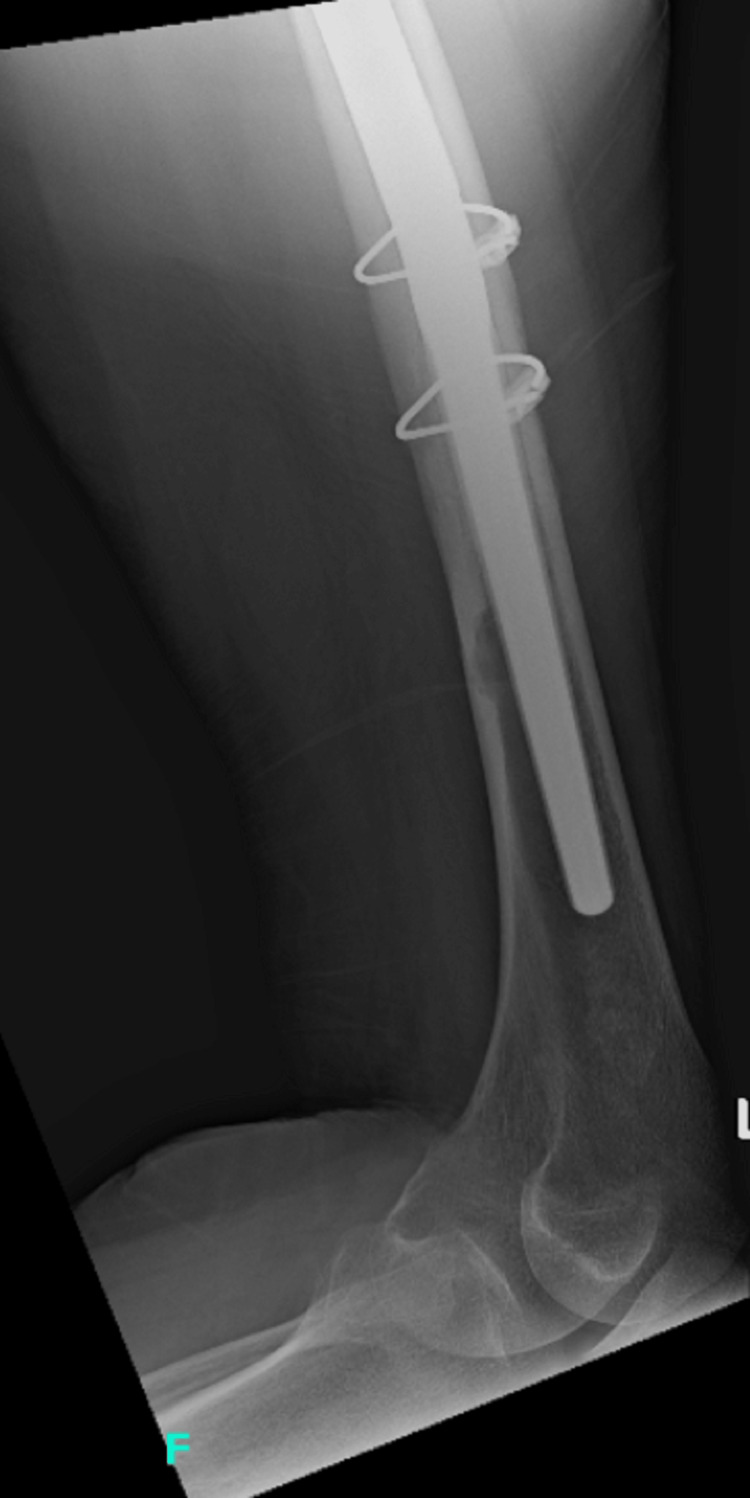
Lateral left femur at three-month follow-up

The patient had an uneventful postoperative course, resumed her chemotherapy and Fosamax regimen, and was discharged on a postoperative day four after she was cleared by the physical therapy team. She was given posterior hip precautions and instructed to weight bear as tolerated, with scheduled outpatient follow-up. She did not undergo any perioperative radiation. At her one-month follow-up, the patient presented in the clinic ambulating with a walker with well-healing incisions. She endorsed occasional pain and mild swelling of the left lower extremity, but her pain had significantly improved from her preoperative status. At the three-month follow-up, she continued to ambulate with a walker and stated that she was getting back to her usual daily activities. Imaging obtained in the clinic at both the one and three-month postoperative appointments yielded a stable appearing left THA with an extended femoral stem, diaphyseal cerclage wires, and greater trochanteric claw plate, all in appropriate and unchanged position and alignment (Figures 13-16).

## Discussion

In light of the current literature and patient case described above, we believe prophylactic fixation, in conjunction with chemotherapy and bone remodeling agents, is a very valuable treatment option for preventing pathologic fractures in MM patients. The use of an extended modular femoral revision system should be strongly considered for patients with pathologic femoral neck fractures who also meet the criteria for prophylactic fixation of the distal femur and peritrochanteric region. In patients without metastatic bone disease, hemi or THA is the mainstay of treatment for displaced femoral neck fractures. With healthy metaphyseal bone in the peritrochanteric region, bony ingrowth of the femoral stem is easily achievable. However, in patients with peritrochanteric lesions, metaphyseal ingrowth of the femoral stem is much less reliable, and the implant is at a higher risk of loosening and failure. In cases such as these, diaphyseal fixation of the femoral stem is paramount. An extended modular femoral system offers an implant option that provides diaphyseal fixation of the femoral stem when metaphyseal fixation is unreliable and was found to be a successful option in the presented case. There is always potential for intraoperative femur fracture during reaming, broaching, or implantation while performing THA, especially in patients with abnormal bone architecture, i.e., patients with MM or metastatic bone disease. To avoid this complication in the future, a smaller stem can be implanted (if amenable) or additional reaming performed if the final impaction of the prosthesis does not advance in a relatively seamless manner. Regardless, great care should be taken when performing this procedure on patients with more fragile bones, especially during impaction.

The use of an extended femoral stem in this patient was also able to provide prophylactic fixation of distal femur lesions. It is known that prophylactically fixing impending fractures in patients with the metastatic bone disease helps prevent future fractures, which decreases a patient’s risk of mortality [6]. Fixation also allows for earlier ambulation, decreased pain, and improved mobility postoperatively [7-9]. Although prophylactic fixation of the femoral shaft is typically achieved with intramedullary nailing, this patient’s multiple lesions in the proximal femur dictated the need for a left THA, negating any benefit of isolated intramedullary fixation. Reinforcing the entire femur was critical in this patient to prevent future femur fracture(s) in the presence of distal femoral lesions.

Given this patient’s younger age and the presence of high bone quality throughout the femur, an uncemented, porous-coated stem was used. Although the data surrounding the use of cemented versus press-fit stems in oncologic patients are mostly neutral [10-12], this case demonstrates the successful use of an uncemented stem in a patient with adequate bone stock. In medically compromised patients, the use of an uncemented stem theoretically reduces surgical time, avoids risks associated with cement pressurization (hypotension, hypoxia, arrhythmias, etc.), and will allow for a much easier procedure should the patient require any revision procedures in the future. In this case, this patient’s high bone quality and level of activity lead us to expect effective ingrowth between the prosthesis and femoral shaft. However, for patients with poor bone quality or patients undergoing radiation postoperatively, cementation will provide reliable fixation of implants without the need for bony ongrowth/ingrowth. Cement also theoretically reduces the occurrence of implant loosening, reducing the need for future procedures in medically compromised patients.

The presence of peritrochanteric lesions and impending trochanteric fracture in this patient warranted prophylactic fixation of the greater trochanter. Reinforcement of the greater trochanter is essential for maintaining the patient's range of motion and mobility [13] and can be easily achieved with plating. A lateral claw plate was placed on the greater trochanter and a single trochanteric bolt with two cerclage cables was used to fix the claw plate to the femoral stem, creating a single, linked construct and providing additional fixation and reinforcement to the left hip. The additional support provided to the greater trochanter by the claw plate will reinforce the area in an effort to prevent future fractures in the area.

Lastly, this patient's chemotherapy regimen was continued up until the time of surgery and was resumed before discharge. There is theoretical concern for poor wound and bone healing when chemotherapy is resumed so soon after surgery, especially with agents like anastrozole commonly used in the treatment of breast cancer. Aromatase inhibitors, such as anastrozole, have proven to lead to bone demineralization and decreased cutaneous wound healing [14,15]. To prevent postoperative complications, it is generally recommended to resume chemotherapy between 30 and 60 days after surgery to allow time for proper healing and recovery [16-18]. However, given this patient's long history of breast cancer and MM, which has now led to two pathologic fractures, it was decided to resume her chemotherapy immediately to prevent any acute progression of either disease. Resuming her chemotherapy so soon after surgery has proven to not be an issue for this patient as she had no wound complications and all follow-up imaging has shown progressive healing and bone formation since surgery.

## Conclusions

The patient case described here and the literature currently available would suggest that the use of an extended femoral stem and greater trochanteric claw plate should be viewed as a viable option in patients requiring THA in the presence of pathologic femoral neck fractures with peritrochanteric and distal femur lesions. This case supports the successful use of an uncemented, press-fit stem, in patients with adequate bone stock where bony ingrowth is reliable, to reduce anesthesia time and avoid complications associated with cemented implants. A claw plate can also be utilized in oncologic patients to provide additional stability to the construct and prophylactically stabilize the greater trochanter. There is room for additional research to be done regarding prophylactic fixation of the femoral shaft and peritrochanteric region in the setting of femoral neck fractures, as well as the use of uncemented femoral stems in oncologic patients with MM.
